# Deleterious Effects of Ethanol, Δ(9)-Tetrahydrocannabinol (THC), and Their Combination on the Spatial Memory and Cognitive Flexibility in Adolescent and Adult Male Rats in the Barnes Maze Task

**DOI:** 10.3390/pharmaceutics12070654

**Published:** 2020-07-09

**Authors:** Ewa Gibula-Tarlowska, Karolina Wydra, Jolanta H. Kotlinska

**Affiliations:** 1 Department of Pharmacology and Pharmacodynamics, Medical University, 20-093 Lublin, Poland; jolanta.kotlinska@umlub.pl; 2 Department of Drug Addiction Pharmacology, Maj Institute of Pharmacology Polish Academy of Sciences, 31-343 Krakow, Poland; wydra@if-pan.krakow.pl

**Keywords:** ethanol, THC, spatial learning, rats

## Abstract

Research demonstrates that adolescents differ from adults in their response to drugs of abuse. The aim of the present study was to examine the influence of ethanol, Δ9-tetrahydrocannabinol hydrochloride (THC), and a combination of these drugs given during adolescence on spatial memory in adolescent and adult rats. Thus, adolescent rats (postnatal day (PND) 30) were subjected to the following groups: 0.9% NaCl; 1.5 g/kg ethanol; 1.0 mg/kg THC; 1.5 g/kg ethanol + 1.0 mg/kg THC. Rats received drug injection four times at three-day intervals. One day after the last injection, half of the treated animals were tested in the Barnes maze task, whereas the remaining animals were tested on PND 70. Results show that there was a significant age effect on spatial memory in the Barnes maze task after these drug administrations. Adolescent animals demonstrated more potent deficits in the spatial learning and memory (probe trial) and in cognitive flexibility (reversal learning) than did adults. However, in adult rats that received these drugs in adolescence, memory decline was observed only after ethanol and ethanol + THC administration. Thus, our results are important in understanding the deleterious impact of THC and/or ethanol abuse during adolescence on memory function across the lifespan (adolescent versus adult).

## 1. Introduction

Adolescence is a period identified by a relatively high occurrence of substance co-use, with alcohol and cannabis (marijuana) as the most frequently used drugs [1]. Combined addiction to ethanol and cannabinoids is an extremely important social and medical problem, particularly as it expands the risk of ethanol dependence in adulthood [2,3]. Likewise, a growing legalization of cannabis combined with decreased perception of harm makes it extremely important to determine the cognitive and behavioral consequences of frequent adolescent exposure to Δ9-tetrahydrocannabinol (THC), the principle psychoactive cannabinoid of marijuana.

In fact, THC is responsible for cognitive dysfunctions, difficulties in learning and memory, psychotic disorders, or “amotivational” syndrome especially in adolescents [4,5,6]. Moreover, prolonged use of THC may lead to withdrawal syndrome characterized by a lack of willingness, apathy, shallow sensations, decline or even disappearance of interests, loss of ambition, and deterioration of the quality of work. Additionally, it may result in impairment of short- and long-term memory or induce difficulties in learning without influence on existing memories or stored information [7,8]. Differences in the development of cognitive impairments are associated with the time of use, as well as the age of users; thus, cannabinoids affect adolescents and adults differently [9]. Moreover, behavioral experiments show that repeated injections of THC in adolescent rats produce a long-lasting response in adulthood, along with a decrease in body weight [10], increased potential of heroin abuse [11], and reinstatement [12] or greater sensitivity to THC-induced learning impairments [13].

One of the most significant cognitive effects of THC is the impairment of learning and memory, mainly in a different paradigm that includes hippocampal function [14,15,16,17]. However, Schaeffer et al. [18] saw cognitive impairments only after heavy exposure (60–110 mg per day) and prolonged use of THC. These were observed as impaired language, memory, multimodal learning, and intellectual functions. In contrast to previous studies, Suliman et al. [19] noted that administration of THC may enhance the concentration of markers involved in all stages of neurogenesis, especially in the hippocampus; thus, its administration might improve the cognitive function of rats. However, these conflicting results need further research since the amnesic effects of THC are not yet systematically studied in animals at different stages of postnatal development.

Similarly, although the effects of ethanol on the central nervous system were extensively studied in adult rodents, these results cannot be translated directly into animals during puberty. Likewise, recent studies showed that ethanol affects brain function differently during adolescence than adulthood. Adolescent rats are more sensitive to the memory-impairing effects and ethanol-induced hypothermia than adults [20,21,22], but they are less sensitive than adults to the effects of ethanol on sedation [23], motor impairment, and dysphoria [24]. This combination of different sensitivities may provoke a state in which adolescents drink more ethanol compared to adults and, consequently, achieve much higher (and more dangerous) blood alcohol concentrations before becoming incapacitated. The higher ethanol levels that are achieved in a maturing brain increase the adolescent’s risk for neurotoxicity and memory impairments. Additionally, not only long-term, but also single administration of ethanol evokes broadly defined memory impairments, deficits, and behavioral disorders [25,26,27]. However, at present, only limited data concerning the long-lasting effects of chronic ethanol exposure during adolescence are available. This is particularly important since both the drinking pattern of adolescents or adults and the neurogenesis processes in these groups differ significantly. Of relevance is the fact that most adolescents drink repeatedly in a chronic, intermittent (“binge”) pattern and this may result in increased risk of neurotoxicity [28,29], as well as impairments of learning [30] and motor function [20,31]. Moreover, it potentiates the risk of addiction development in the future.

The fact that both ethanol and THC impair learning is obviously of great importance, particularly since, among adolescents, ethanol and marijuana are often used in combination. While the effects of ethanol and cannabinoids on learning and memory processes are extensively documented in adults, there are many contradictory studies on the effects of these substances on individuals in adolescence. In addition, only limited data about their simultaneous use on cognitive functions in adulthood are reported so far.

Although ethanol is known and abused for hundreds of years, it still remains the subject of study, and the exact mechanism via which it acts is not clearly established. So far, no specific receptors for ethanol are identified; however, ethanol affects multiple receptors and neurotransmitters, e.g., endogenous opioid peptides, dopamine, and serotonin. In addition, several studies provided evidence for participation of cannabinoid type 1 (CB1) receptors in the pharmacological actions of ethanol [32,33,34]. Nevertheless, ethanol-induced dopamine release is partially associated with the stimulation of CB1 receptors [35] that may potentiate the ethanol-enhancing effects when co-administered with cannabinoids. These observations suggest that cannabis or THC administration may have a significant impact on ethanol activity. Therefore, the aim of our study, using the Barnes maze task, was to reveal whether THC and/or ethanol, after administration during adolescence, induced greater spatial memory impairment in adolescent (postnatal day (PND) 40) than adult (PND 70) male rats, when compared to these drugs alone.

## 2. Materials and Methods

### 2.1. Animals

The experiment was conducted on adolescent (PND 30) and adult (PND 70) male Wistar rats (supplied through in-house breeding by the Center for Experimental Medicine in Lublin, Poland). They were housed under standard laboratory conditions (22 °C, 12-h:12-h light/dark cycle) in groups of five rats per cage, with standard food (Bacutil, Motycz, Poland) and water ad libitum. The rats were adapted to the laboratory conditions and were handled for at least one week before the experiments. In all experiments, the same groups of animals were used (*n* = 8–10). All experimental protocols and housing conditions were approved by the Local Ethics Committee and were carried out according to the National Institute of Health Guidelines for the Care and Use of Laboratory Animals, as well as the European Community Council Directive of November 2010 for the Care and Use of Laboratory Animals (Directive 2010/63/EU), and they were approved by the Local Ethics Committee.

### 2.2. Drugs

**Figure 1:**
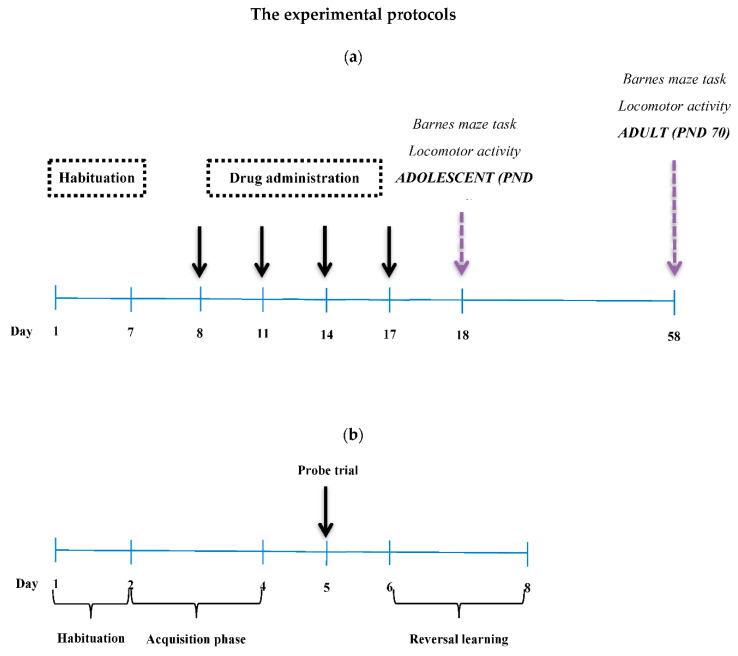
Diagram of experimental design. (**a**) The experimental protocol; (**b**) The phases of Barnes maze task.

THC (LGC Standards, Poland) was prepared in a mixture of propylene glycol (Sigma Aldrich, Germany) and Tween-80 (Sigma Aldrich, Germany) (1:1, *v*/*v*) and stored at −20 °C until used. A working solution was prepared before experiment by dissolving stock solution in sterile saline (0.9% NaCl). THC (1.0 mg/kg) was administrated as an intraperitoneal (i.p.) injection at a volume of 1 mL/kg for rats. Ethanol (95% *w*/*v*, Polmos, Poznan, Poland) was diluted with 0.9% NaCl to the concentration 15% *w*/*v* and administrated i.p. at the dose of 1.5 g/kg. In the current study, the method, including drug dosage regimens, described by Swartzwelder et al. [22] was implemented for determining the effects of THC and ethanol on learning and memory. The doses were selected based on prior work that demonstrated an impairment effect on spatial learning of ethanol doses of 1 g/kg and 2 g/kg in adolescent, but not in adult rats [20]. The THC dose of 1.0 mg/kg was chosen based on a previous study [36,37] and reports from human literature suggesting that co-administration of ethanol and THC may result in increased plasma THC levels, thereby increasing the effective dose of THC [38]. After habituation to the laboratory conditions (seven days), at postnatal day (PND) 30, animals were categorized into four groups (vehicle, 1.5 g/kg ethanol, 1.0 mg/kg THC, and 1.5 g/kg EtOH + 1.0 g/kg THC), each receiving substances four times at 72-h intervals. The order of drug treatment conditions was counterbalanced across test sessions. Then, 24 h after the last injection, half of the animals in each group were subjected to experiments (adolescent groups). The other half of the animals were returned to their home cages and housed until PND 70 when they, in turn, were subjected to experiments (adult groups). Thus, adolescent animals were PND 40 and adult animals were PND 70 at the beginning of the experiments (Figure 1a).

#### 2.2.1. Barnes Maze Task

Barnes circular maze (Stoelting, Dublin, Ireland) is a gray metal platform with a diameter of 122 cm and a height of 90 cm. On the perimeter of the platform, 20 holes are placed with a diameter of 10 cm each, where only one is the entrance to an under-platform shelter chamber with dimensions of 12 × 12 × 35 cm—referred to as an escape box. In the task, the animal is placed in the middle of the platform and is initially unable to locate the escape box, the location of which can vary according to the phase of the task. Additional stimuli are provided during the task. One is in the form of intense lighting—two points of light placed 1.5 m above the platform with a power of 500 W each. The other stimulus is a loud buzzer sound of 80 dB. Additionally, on the walls of the laboratory room, visual cues are provided in the form of large colorful geometric figures and signs placed to facilitate the location of the escape box by the animal [39]. The Barnes maze task consists of the following phases: habituation (one day), acquisition phase (three days), probe trial (one day), and reversal learning (three days) (Figure 1b). The experimental design was developed based on the methods used previously by other authors (see References [39,40,41]).

#### 2.2.2. Horizontal Locomotor Activity Test

The locomotor activity of rats was measured using a photocell apparatus (Porfex, Bialystok, Poland). The animals were placed individually in 60 × 60 cm transparent Plexiglas boxes. The boxes were equipped with infrared sensors placed at 45 and 100 mm above the floor. Locomotor activity was recorded as horizontal activity (total distance traveled (m)) for a period of 15 min. The test was carried out in a soundproof room with the lights turned off. This test was performed after the probe trial on PND 40.

#### 2.2.3. Experiment 1

The effect of adolescent ethanol, THC, or the exposure of both on spatial memory acquisition in adolescent and adult rats in the Barnes maze task was studied in Experiment 1.

The experiment began at PND 30 for adolescent and PND 70 for adult rats. One day before the acquisition phase, the rats were habituated (one day) to the platform to reduce anxiety behavior (to familiarize rats with the maze and the escape box). The animals were placed in the middle of the platform and were allowed to freely explore the apparatus for 180 s. Following habituation, rats completed acquisition training for three consecutive days (one training session with three trials per day). On each trial, the rats were given 180 s to enter the escape box. The number of errors made (head-dips into incorrect hole) and latency to find the escape box were recorded in each trial to assess learning. During acquisition, the animals did not receive any substance.

#### 2.2.4. Experiment 2

The effect of adolescent ethanol, THC, or the exposure of both on spatial memory retrieval in adolescent and adult rats in the Barnes maze task was studied in Experiment 2.

The probe trial was performed for adolescent and adult rats 24 h after the end of the three days of acquisition sessions. During this trial, no escape box was present. Memory of the location of the escape box was assessed for 90 s. The primary latency and primary errors to reach the location of the escape box were counted. Herein, rats did not receive any substances.

#### 2.2.5. Experiment 3

The effect of adolescent ethanol, THC, or the exposure of both on cognitive flexibility in adolescent and adult rats in the Barnes maze task was studied in Experiment 3.

The reversal learning for adolescent and adult rats was performed 24 h after the end of the probe trial. At that time, the position of the escape box was rotated 180° in relation to the original, and two 180-s reversal learning trials were run for three consecutive days. During the reversal test, the time for entry of the animal into the escape box was recorded, and the number of errors made was counted.

#### 2.2.6. Experiment 4

The effect of adolescent ethanol, THC, or the exposure of both on locomotor activity in adolescent and adult rats was studied in Experiment 4.

The experiment began at PND 40 for adolescent and PND 70 for adult rats. The individual rats were placed in cages, and horizontal activity (total distance traveled) was measured for 15 min. After each session, the cages were washed with 10% *w*/*v* ethanol to dissipate odor cues.

### 2.3. Statistical Analysis

The obtained results were collected and developed using the Graph-Pad Prism 5.03 computer program. Statistical analysis of test results was based on two-way analysis (ANOVA) using the factors of treatment and days) or treatment and age. This was followed by the Bonferroni test. The results were presented as mean ± standard error (SEM). A *p*-value less than 0.05 (*p* < 0.05) was considered statistically significant for all tests.

## 3. Results

### 3.1. Experiment 1

The effect of adolescent ethanol, THC, or the exposure of both on spatial memory acquisition in adolescent and adult rats in the Barnes maze task was studied in Experiment 1.

**Figure 2:**
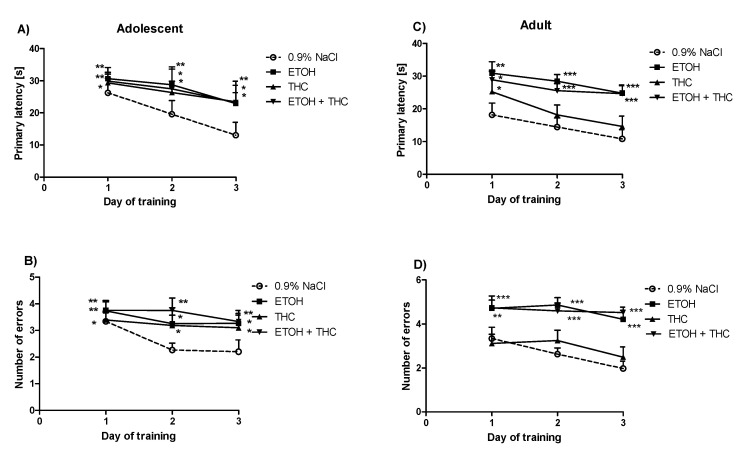
The deleterious effect of ethanol, Δ9-tetrahydrocannabinol (THC), or the administration of both during adolescence on spatial memory acquisition in adolescent and adult rats in the Barnes maze task. * *p* < 0.05, ** *p* < 0.01, *** *p* < 0.001 vs. 0.9% NaCl (adolescent or adult rats). Primary latency (panels **A**, **C**) and number of errors (panels **B**, **D**) measured during memory acquisition shown as mean ± SEM of the three trials.

The Barnes maze task was carried out for adolescent rats 24 h after the last administration of psychoactive substances. In the primary latency, a two-way ANOVA with repeated measures showed the statistically significant effect of day of training (F(2,104) = 3.04, *p* < 0.05) (Figure 2A). The post hoc test (Bonferroni test) revealed that exposure to psychotropic substances during adolescence impairs memory processes in adolescent rats. This effect was observed as an increase in the primary latency on the first, second, and third days of training in the group pre-exposed with ethanol (*p* < 0.01), THC (*p* < 0.01), and a combination of ethanol and THC (*p* < 0.01, *p* < 0.05, and *p* < 0.05, respectively) compared to animals from the control group (0.9% NaCl) (Figure 2A). Moreover, in the number of primary errors, the two-way ANOVA with repeated measures uncovered the significant effect of treatment in adolescent rats (F(3,104) = 3.34, *p* < 0.05). The Bonferroni post hoc test showed an increase in the number of primary errors committed by animals exposed to ethanol on the first (*p* < 0.01), second (*p* < 0.05), and third (*p* < 0.05) days of acquisition. Finally, an increase in the number of primary errors committed by the group exposed to THC (*p* < 0.05) and to a combination of ethanol and THC (*p* < 0.01) was observed in all days of acquisition compared to the control group (Figure 2B).

The Barnes maze task for adults was carried out on PND 70. In the primary latency, a two-way ANOVA with repeated measures showed the statistically significant effect of day of training (F(2,104) = 4.65, *p* < 0.01) and treatment (F(3,104) = 10.83, *p* < 0.01) (Figure 2C). In addition, the post hoc test (Bonferroni test) revealed that the administration of psychotropic substances during adolescence impairs learning in adult rats exposed to these substances. This effect was observed as an increase in the primary latency on the first (*p* < 0.01), second (*p* < 0.001), and third (*p* < 0.001) days of acquisition in the group of adult rats exposed to ethanol, on the first (*p* < 0.05) day of training in the group of adult rats exposed to THC (*p* < 0.01), and on the first (*p* < 0.05), second, and third (*p* < 0.001) days in the group of adult rats exposed to a combination of ethanol and THC (Figure 2C). In the number of primary errors, a two-way ANOVA with repeated measures demonstrated the statistically significant effect of treatment (F(3,104) = 20.27, *p* < 0.001). The post hoc test (Bonferroni test) brought to light the increased number of primary errors committed by adult rats exposed to ethanol on all days of training (*p* < 0.001). Likewise, exposure to a combination of ethanol and THC during adolescence resulted in adult rats committing an increased number of primary errors on the first (*p* < 0.01), second (*p* < 0.001), and third (*p* < 0.001) days of training (Figure 2D).

### 3.2. Experiment 2

The effect of adolescent ethanol, THC, or the exposure of both on spatial memory retrieval in adolescent and adult rats in the Barnes maze task was studied in Experiment 2.

**Figure 3:**
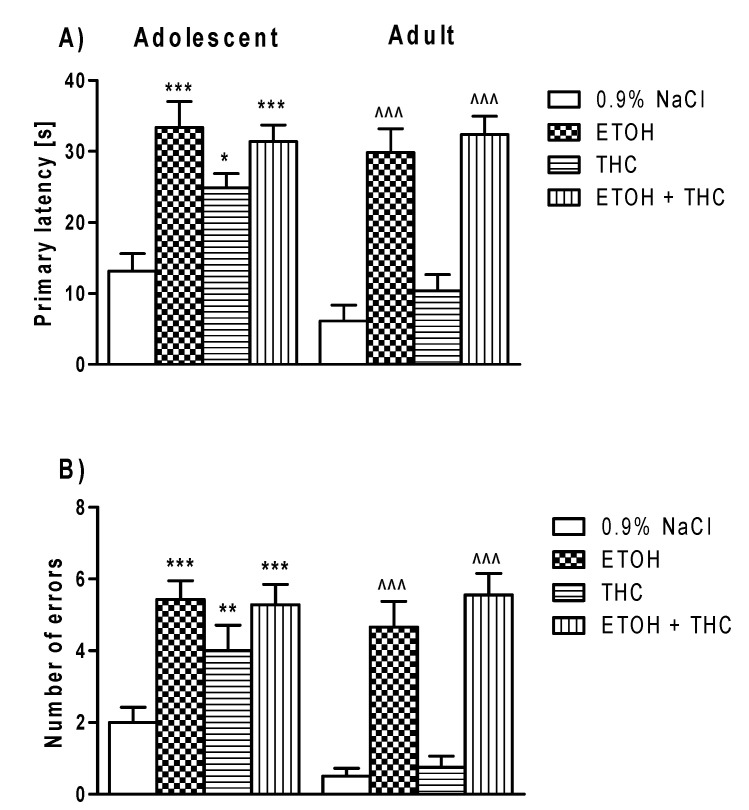
The deleterious effect of ethanol, THC, or the administration of both during adolescence on spatial memory retrieval in adolescent and adult rats in the Barnes maze task. * *p* < 0.05, ** *p* < 0.01, *** *p* < 0.001 vs. 0.9% NaCl; ^^^ *p* < 0.001 vs. 0.9% NaCl (adolescent or adult rats). Primary latency (panels **A**) and number of errors (panels **B**), measured during the probe trial are shown as mean ± SEM

In this experiment, 24 h after the acquisition phase, spatial memory was evaluated by probe trial. The analysis of two-way ANOVA indicated statistically significant differences between the tested groups of adolescent rats exposed to psychoactive substances in primary latency (effect of treatment F(3,70) = 5.99, *p* < 0.001; effect of age F(1,70) = 17.57, *p* < 0.001) (Figure 3A) and the number of committed primary errors (effect of treatment F(3,70) = 10.6; *p* < 0.001; effect of age F(1,70) = 26.54; *p* < 0.001) (Figure 3B).

The post hoc test (Bonferroni test) showed that treatment with ethanol, THC, and both psychoactive substances together interfered with the adolescent rat memory processes on the test day (probe trial). This effect was observed as an increase in the primary latency of rats pre-treated with ethanol (*p* < 0.001), THC (*p* < 0.05), and a combination of ethanol and THC (*p* < 0.001) (Figure 3A). The impairment of memory in the Barnes maze task was also observed as an increase in the number of primary errors committed by adolescent rats exposed to ethanol (*p* < 0.001), THC (*p* < 0.01), and a combination of ethanol and THC (*p* < 0.001) (Figure 3B).

Moreover, the post hoc test (Bonferroni test) revealed that treatment with ethanol, THC, and a combination of both psychoactive substances during adolescence interfered with memory processes in adult rats on test day (probe trial). This effect was observed as an increase in the primary latency in the ethanol (*p* < 0.001) and the combination of ethanol and THC (*p* < 0.001) groups, as compared to control adult rats (Figure 3A). The impairment of memory in the Barnes maze test was also observed as an increase in the number of primary errors committed by rats treated with ethanol (*p* < 0.001) and with a combination of ethanol and THC (*p* < 0.001) (Figure 3B).

### 3.3. Experiment 3

The effect of adolescent ethanol, THC, or the exposure of both on cognitive flexibility in adolescent and adult rats in the Barnes maze task was studied in Experiment 3.

**Figure 4:**
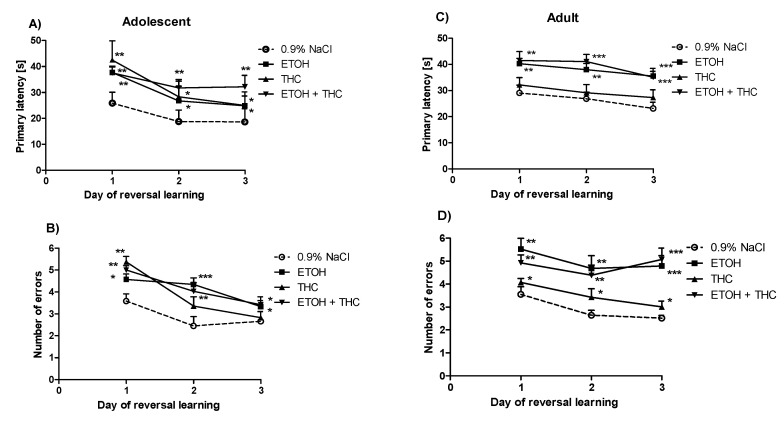
The deleterious effect of ethanol, THC, or the administration of both during adolescence on cognitive flexibility in adolescent and adult rats in the Barnes maze task. * *p* < 0.05, ** *p* < 0.01, *** *p* < 0.001 vs. 0.9% NaCl (adolescent or adult rats). Primary latency (panels **A**, **C**) and number of errors (panels **B**, **D**) measured during reversal learning shown as mean ± SEM of the two trials.

The analysis of two-way ANOVA showed statistically significant differences in reversal learning trials between the tested groups of adolescent animals exposed to psychoactive substances, in primary latency (effect of treatment F(3,104) = 4.21, *p* < 0.001; effect of day of reversal learning F(2,104) = 4.16, *p* < 0.05) (Figure 4A) and in the number of primary errors (effect of treatment F(3,104) = 6.65, *p* < 0.001; effect of day F(2,104) = 22.82, *p* < 0.001) (Figure 4B). The post hoc test Bonferroni demonstrated that psychoactive substances given to rats during adolescence impaired the cognitive flexibility of these rats in the Barnes maze task. This effect was observed as an increase of the primary latency for animals that received ethanol during adolescence on the first (*p* < 0.01), second, and third days (*p* < 0.05) of trials in reaching the target hole. Moreover, in rats that received THC and a combination of ethanol and THC during adolescence, the primary latency was increased on the first (*p* < 0.01), second (*p* < 0.001 and *p* < 0.01, respectively), and third days (*p* < 0.05 and *p* < 0.01, respectively) of trials in reaching the target hole (Figure 4A). Furthermore, the Bonferroni test revealed the increased number of primary errors committed on the first, second, and third (*p* < 0.05) days of trials by the group of rats that received ethanol during adolescence. Similarly, the administration of THC during adolescence increased the number of primary errors on the first day (*p* < 0.01), while the combined administration of ethanol and THC during adolescence did so on the first (*p* < 0.01), second (*p* < 0.01), and third (*p* < 0.05) days of the reversal learning for adolescent animals (Figure 4B).

In the primary latency for adult rats, a two-way ANOVA with repeated measures showed the significant effect of treatment (F(3,104) = 15.11, *p* < 0.001) and day of reversal learning (F(2,104) = 3.97, *p* < 0.001) (Figure 4C). The two-way ANOVA with repeated measures also revealed the statistically significant effect of treatment (F(3,104) = 22.4, *p* < 0.001) and day of reversal learning (F(2,104) = 4.91, *p* < 0.001) on the number of primary errors committed before reaching the target hole (Figure 4D). The post hoc test Bonferroni brought to light that ethanol administered to rats during adolescence impaired the cognitive flexibility of these rats when measured during adulthood. This effect was observed as a significant increase in the primary latency on all days of reversal learning in the group of adult rats that received ethanol and a combination of ethanol (*p* < 0.01, *p* < 0.01, and *p* < 0.001) and a combination of ethanol and THC (*p* < 0.01, *p* < 0.001, and *p* < 0.001) during adolescence (Figure 4C). Moreover, an increase in the number of primary errors was observed on the first, second, and third days of reversal learning by groups of adult rats pre-treated by ethanol (*p* < 0.01, *p* < 0.01, and *p* < 0.001), THC (*p* < 0.05), or both substances (*p* < 0.01, *p* < 0.01, and *p* < 0.001) during adolescence (Figure 4D).

### 3.4. Experiment 4

The effect of adolescent ethanol, THC, or the exposure of both on locomotor activity in adolescent and adult rats was studied in Experiment 4.

**Figure 5:**
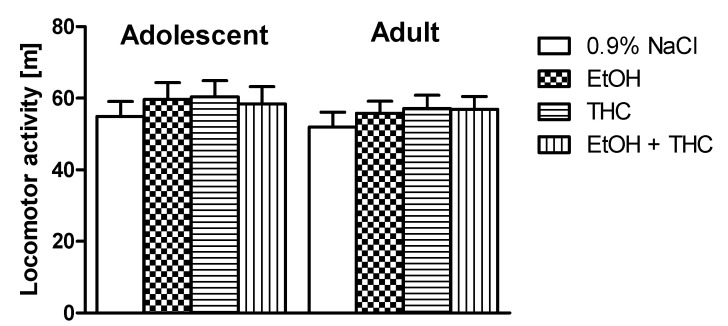
The effect of ethanol, THC, or the administration of both during adolescence on locomotor activity (total distance traveled) in adolescent and adult rats.

Analysis of two-way ANOVA did not show a statistically significant effect in age or treatment on the total distance traveled by rats (effect of treatment F (3,70) = 0.58, *p* > 0.05; effect of age F (1,70) = 0, *p* > 0.05; interactions F (3,70) = 0, *p* > 0.05) (Figure 5).

## 4. Discussion

Our results confirmed the deleterious effects of THC and/or ethanol after administration during adolescence on learning and memory processes. We demonstrated that THC, as well as ethanol and their combination, administrated four times at 720 h intervals, from 30 PND, had a detrimental impact on the behavior of adolescent rats in the Barnes maze task. In our experiments, all these psychoactive substances produced impairment of both spatial learning and cognitive plasticity of adolescent rats. The most pronounced effect was observed in the groups exposed to ethanol or combination of ethanol and THC. In contrast, insignificant or absence of spatial learning and learning plasticity impairments were observed for adult rats (70 PND) exposed to THC in adolescence. Additionally, the locomotor activity test did not demonstrate statistically significant differences between the control group, receiving 0.9% NaCl and animals receiving psychoactive substances in both adolescence and adult groups. Therefore, the obtained results excluded the impact of locomotor activity on the behavior of animals in the Barnes maze task.

In fact, our experiments revealed comparable impairments of learning during the acquisition phase in all groups of adolescents who were exposed to psychoactive substances. In contrast, Nelson et al. [42] did not show any impairment in spatial learning and memory in a Barnes maze in adolescent male rats who were co-exposed to ethanol and subcutaneous or oral THC. Several factors, however, may have an influence on such diverse outcomes. One is the coincident peak blood THC and alcohol concentration that may be subthreshold but is required to uncover interactive effects between THC and moderate alcohol intake [43]. Furthermore, THC is only a partial CB1 receptor agonist. Indeed, cognitive deficits occurred when potent CB1 receptor agonists such as WIN 55,212-2 and CP 55,940 were used [44,45]. Nevertheless, in our study, the THC exposure did induce impairments in spatial learning in adolescent rats, although to a lesser extent than the administration of ethanol or a combination of these substances.

The mechanism of such ethanol–THC interactions is unknown. However, published data show that cannabinoids increase the susceptibility of the immature brain to ethanol neurotoxicity. Probably, CB1 receptor activation modulates gamma-aminobutyric acid (GABA)-ergic and glutamatergic neurotransmission and primes the developing brain to suffer apoptotic neuronal death [46]. Ethanol, similar to *N*-methyl-d-aspartate (NMDA) glutamate receptor antagonists and GABAA receptor agonists, induces neuronal death in the developing brain by suppressing synaptic activity, which, at this age, elicits vital trophic signals for immature neurons [47]. It should also be noted that both ethanol [48,49] and THC [50] are mitochondrial toxins, especially at higher doses, and this effect could lead to neuronal dysfunction and deficits in cognitive performance.

Although previous studies examined the acute behavioral effects of cannabinoids in rodents, only a few concerned their lasting effects at different developmental ages. Prior experiments with rodents documented cognitive impairment [14,51,52,53,54,55], lack of effects [56], or even enhanced spatial memory [57] after THC administration. Furthermore, some studies indicated that the endocannabinoid system plays a neuroprotective role [58,59,60,61]. Nevertheless, despite THC being mainly used by adolescents, there are few studies comparing its impact on the behavior of adolescents and adults after administration in this developmental age.

The absence of learning impairments in adult rats exposed to THC in our experiments is consistent with evidence from other behavioral studies where it was found that the adverse effects of THC or the cannabinoid receptor agonist, WIN 55,212-2, on learning are more severe in adolescents than in adults [8,56,62,63]. Cha et al. [56] found that subchronic THC treatment disrupted learning in both adolescents and adults, but the significance of this effect was dependent upon both the age and sex of animals. However, for most of these experiments, THC was administered to either adolescent or adult animals. In contrast, in our experiments, cannabinoids were administrated only in adolescence, and their effects were determined in adolescence and in adulthood. As previously reported, our results reveal that exposure to THC attenuates spatial memory acquisition in adolescent rats and only slightly affects the behavior of these animals in adulthood. Therefore, we presume that exposure to THC may be particularly detrimental during adolescence. Nevertheless, the precise mechanisms underlying the THC effects on learning are not yet fully understood. The theory explaining this effect is inhibition of glutamate release [64] (as mentioned above) or regulation of synaptic plasticity by cannabinoids [65].

Others suggest that cannabinoids may affect functioning of the crucial memory-relevant brain areas by influencing the homeostasis of the endocannabinoid system. In fact, the hippocampus structure is important for spatial learning and memory as it holds the highest density of CB1 receptors, especially in adolescent animals. Thus, it is particularly susceptible, and this situation accounts for the disruptive effects of THC on memory [66,67]. Likewise, it was suggested that adolescence represents a period of increased vulnerability to neural changes induced by THC, resulting in long-lasting or even permanent deficits in cognitive function. However, our results did not confirm long-lasting impairment of spatial memory in adult rats after exposure to THC in adolescence. Presumably, the occurrence of the maximum level of CB1 receptors is in early adolescence and its decline with the age of the animal has an effect upon the degree of higher learning impairment in the group of adolescent rats, while this situation may decrease with age [68]. Thus, the outcome of our experiment may also be affected by the regimen of administration of THC, since, in previous studies, long-lasting memory deficits were observed after longer periods of administration than used in our study [69,70,71,72].

Our results are consistent with other experiments that used higher doses of THC [73,74], and a period of abstinence after adolescent exposure [75] did not indicate robust alternations in adult behavior. This suggests that adverse effects associated with adolescent THC use might be due to the non-cannabinoid concomitants of cannabis use. Therefore, we conclude that the degree and duration of learning impairments after THC exposure depend on the age of the tested animals, as well as the intensity and period of THC administration. The differences in the mechanism of action of ethanol and THC are crucial. THC acts mainly through CB1 receptors, while ethanol affects many receptors and neurotransmitters and induces potent and permanent neurotoxic effects.

The results obtained for the group of adult rats, however, indicated long-lasting memory impairments, persisting even up to three weeks after the last administration of ethanol. This outcome is in line with previous observations that clearly suggest the negative effects of exposure to ethanol during adolescence on subsequent behavior, neurobiology, and response to ethanol [76,77]. A potential mechanism involved in the observed effects is the ethanol-induced decrease of brain-derived neurotrophic factor (BDNF) in the hippocampus. This neurotrophic factor acts to regulate development processes, plasticity, and addiction processes. In addition, several studies suggested that a decreased of BDNF activity may be involved in ethanol-induced neurodegeneration and in the etiology of ethanol-related neurodevelopmental disorders. Therefore, as BDNF deficiency, especially in the hippocampus structure, affects the processes of spatial learning and memory, this effect may explain our experimental outcomes [78,79,80]. In addition, the long-lasting impairment that was noted may also be related to the lack of tolerance to the neurotoxic effects of ethanol, as well as permanent damage to the hippocampus area after its administration during adolescence. In fact, adolescence represents a period of heightened vulnerability to repeated ethanol intake. Thus, exposure during this period even only to the extent of recreational drinking during adolescence may lead to enhanced potential for ethanol abuse disorders in adulthood.

In addition, the memory impairment observed in the group previously receiving ethanol and the combination of ethanol and THC was similar in rats at both stages of lifespan. No additive effect was demonstrated, and this outcome clearly indicates that the ethanol administration is primarily responsible for the memory impairment effect even after a prolonged withdrawal period. Moreover, ethanol seems to be a much more potent neurotoxin than THC and probably disturbs neurogenesis during adolescence to larger extent than does THC and, therefore, the effects persist for a long time. In addition, available data clearly indicate that the combined abuse of THC and ethanol in adolescents may increase the risk of cognitive disorders and addiction to these substances [81].

Similarly, in the case of cognitive plasticity, the observed impairments in adolescent rats and in adults were much more pronounced when ethanol was administrated. Our experiments showed that intermittent THC administration in adolescence induced impairments of cognitive plasticity to a lesser extent than did ethanol administration alone or its combination with THC. The obtained results confirmed the available data from the literature, which emphasize that both acute [82] and chronic administration of ethanol cause long-term deficits of memory plasticity [83,84,85,86,87]. In addition, in our experiments, this effect of THC administration was poorly marked in adult animals that were tested at PND 70. Similar effects were previously observed using an aqueous labyrinth in which chronic administration of THC in adolescence did not significantly affect further learning four weeks after the end of administration of this compound [36]. It should be emphasized that spatial learning deficits are attributed to impaired hippocampal function [88], while impairment of reversal learning is related to dysfunctions in the prefrontal cortex [85,89]. Therefore, ethanol appears to induce potent and persistent morphological and functional alterations within hippocampal neurons in the still developing brain and reduces the ability of prefrontal cortex to flexibly modulate behavior during changing environmental situations.

The main limitation of our study is the use of only a single dose of ethanol or THC. However, as we stated before, the doses of ethanol and THC were chosen based on previous study [20,22], taking into account the age-dependent impairing effect of ethanol on memory that is dose-dependent, as well as the ethanol–THC interaction on plasma THC level [38]. It should be mentioned that such combinations of ethanol–THC doses impaired recognition memory and object preference in an age-dependent manner [22]. Another limitation of our study is the use of only male rats. There is literature documenting important differences between male and female adolescent animals with regard to both ethanol and THC effects [56,90]. Advancing the understanding of neurodevelopmental ethanol–THC combination effects will require inclusion of both males and females in such experiments. These are being considered.

Based on our results, we conclude that ethanol, THC, or combined use of ethanol and THC in adolescence significantly increases the risk of developing learning and memory disorders in both adolescent and adult animals. However, these findings emphasize the need for further investigations regarding the role of early-onset cannabis and ethanol use on neurodevelopmental and cognition processes, as well as the effects of sex differences.
